# Dichlorido{*N*′-[1-(2-pyridin-2-yl)ethyl­idene]acetohydrazide-κ^2^ *N*′,*O*}copper(II)

**DOI:** 10.1107/S1600536810053195

**Published:** 2010-12-24

**Authors:** Amitabha Datta, Kuheli Das, Yan-Ming Jhou, Jui-Hsien Huang, Hon Man Lee

**Affiliations:** a National Changhua University of Education, Department of Chemistry, Changhua, Taiwan 50058

## Abstract

In the title compound, [CuCl_2_(C_9_H_11_N_3_O)], the Cu^II^ atom is in a distorted square-pyramidal CuCl_2_N_2_O coordination geometry. The tridentate acetohydrazide ligand chelates in a meridional fashion. The chloride ligand in the axial position forms a long Cu—Cl distance of 2.4892 (9) Å. In contrast, the Cu—Cl distance from the equatorial chloride ligand is much shorter [2.2110 (7) Å]. Inter­molecular N—H⋯Cl and C—H⋯Cl hydrogen bonds link the complexes into a three-dimensional network.

## Related literature

For a related copper(II) complex with a similar tridentate ligand, see: Recio Despaigne *et al.* (2009 ▶).
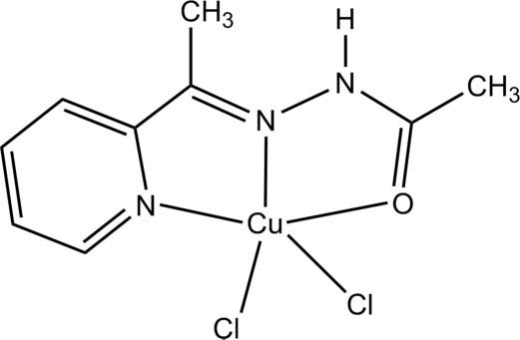


## Experimental

### 

#### Crystal data

[CuCl_2_(C_9_H_11_N_3_O)]
*M* *_r_* = 311.65
Monoclinic, 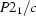

*a* = 6.6501 (15) Å
*b* = 15.680 (3) Å
*c* = 13.103 (2) Å
β = 118.769 (12)°
*V* = 1197.7 (4) Å^3^
*Z* = 4
Mo *K*α radiation
μ = 2.25 mm^−1^
*T* = 150 K
0.25 × 0.20 × 0.19 mm

#### Data collection

Bruker SMART APEXII diffractometer
Absorption correction: multi-scan (*SADABS*; Sheldrick, 2003 ▶) *T*_min_ = 0.603, *T*_max_ = 0.674
16039 measured reflections
3081 independent reflections
2534 reflections with *I* > 2.0σ(*I*)
*R*_int_ = 0.027

#### Refinement

*R*[*F* ^2^ > 2σ(*F* ^2^)] = 0.030
*wR*(*F* ^2^) = 0.075
*S* = 1.02
3081 reflections
147 parameters
H-atom parameters constrained
Δρ_max_ = 0.51 e Å^−3^
Δρ_min_ = −0.54 e Å^−3^

### 

Data collection: *APEX2* (Bruker, 2007 ▶); cell refinement: *SAINT* (Bruker, 2007 ▶); data reduction: *SAINT*; program(s) used to solve structure: *SHELXS97* (Sheldrick, 2008 ▶); program(s) used to refine structure: *SHELXL97* (Sheldrick, 2008 ▶); molecular graphics: *SHELXTL* (Sheldrick, 2008 ▶); software used to prepare material for publication: *DIAMOND* (Brandenburg, 2006 ▶).

## Supplementary Material

Crystal structure: contains datablocks I, global. DOI: 10.1107/S1600536810053195/pv2372sup1.cif

Structure factors: contains datablocks I. DOI: 10.1107/S1600536810053195/pv2372Isup2.hkl

Additional supplementary materials:  crystallographic information; 3D view; checkCIF report

## Figures and Tables

**Table 1:** Hydrogen-bond geometry (Å, °)

*D*—H⋯*A*	*D*—H	H⋯*A*	*D*⋯*A*	*D*—H⋯*A*
N1—H1⋯Cl2^i^	0.89	2.34	3.226 (2)	170
C1—H1*A*⋯Cl1^ii^	0.98	2.63	3.529 (3)	153
C3—H3*A*⋯Cl2^i^	0.98	2.81	3.785 (3)	176
C3—H3*C*⋯Cl1^iii^	0.98	2.75	3.703 (3)	165
C7—H7⋯Cl1^iv^	0.95	2.68	3.529 (3)	149

Symmetry codes: (i) 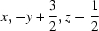; (ii) 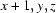; (iii) 
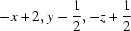
; (iv) 
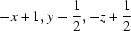
.

**Fig. 1.**
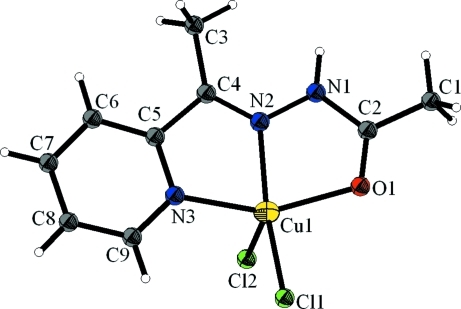
The structure of the title compound, showing 50% probability displacement ellipsoids for the non-hydrogen atoms. The H atoms are dipicted by circles of an arbitrary radius.

**Fig. 2.**
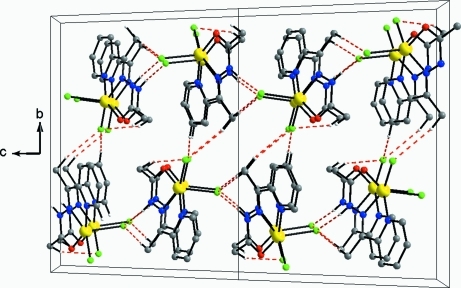
A view of the crystal packing along the a axis, displaying the hydrogen bonds as dashed lines; H-atoms not involved in hydrogen bonding have been excluded.

## supplementary crystallographic information

### Comment

In the title compound (Fig. 1), the copper atom is in distorted square coordination geometry with the ligand, 2-benzoylpyridine-methyl hydrazone (*L*) coordinated in meridional fashion *via* the pyridyl N, imine N, and keto O atoms. The equatorial chloride is *trans* to the imine N. Another chloride ligand occupies the axial position. Interestingly, the two Cu—Cl distances are unequal in length. The chloride ligand in the axial position forms a long Cu—Cl distance of 2.4892 (9) Å. In contrast, the Cu—Cl distance from the equatorial chloride ligand is much shorter (2.2110 (7) Å). The ligand is in keto form as indicated by the short C2—O1 distance of 1.240 (3) Å. Classical intermolecular hydrogen bonds of the type N—H···Cl and non-classical intermolecular hydrogen bonds of the type C—H···Cl link the complexes into a three dimensional network.

The structure of a copper(II) dichloride complex with a similar tridentate hydrazone ligand has been reported in the literature (Recio Despaigne *et al.*, 2009).

### Experimental

The tridenate hydrazone ligand was prepared by the condensation of acetyl hydrazide (0.074 g, 1.0 mmol) with 2-acetylpyridine (0.112 ml, 1.0 mmol) in methanol (15 ml). On refluxing the methanolic solution for 2 h a pale yellow color was observed, an indication of the formation of Schiff base ligand. On removal of the solvent, the resultant light yellow liquid was used without further purification. To a hot methanolic solution (30 ml) of anhydrous CuCl_2_ (0.134 g, 1.0 mmol), the ligand (0.177 g, 1.0 mmol) was added. The solution immediately turned to a green color. Then the mixture was heated to boiling for 10 min. After cooling, it was placed inside a refrigerator. Dark green prismatic crystals were formed in 7 days. The crystals were filtered off, washed with water and dried in air.

### Refinement

All the hydrogen atoms could have been discerned in the difference Fourier map, nevertheless, all the H atoms were positioned geometrically and refined as riding atoms, with C_aryl_—H = 0.95, C_methyl_ —H = 0.98 and NH = 0.89 Å while *U*_iso_(H) = 1.2*U*_eq_(C_methine_ and N) and *U*_iso_(H) = 1.5 *U*_eq_ (C_methyl_).

### Crystal data

**Table:**

[CuCl_2_(C_9_H_11_N_3_O)]	*F*(000) = 628
*M**_r_* = 311.65	*D*_x_ = 1.728 Mg m^−^^3^
Monoclinic, *P*2_1_/*c*	Mo *K*α radiation, λ = 0.71073 Å
Hall symbol: -P 2ybc	Cell parameters from 6047 reflections
*a* = 6.6501 (15) Å	θ = 2.2–27.7°
*b* = 15.680 (3) Å	µ = 2.25 mm^−^^1^
*c* = 13.103 (2) Å	*T* = 150 K
β = 118.769 (12)°	Prism, green
*V* = 1197.7 (4) Å^3^	0.25 × 0.20 × 0.19 mm
*Z* = 4	

### Data collection

**Table:**

Bruker SMART APEXII diffractometer	3081 independent reflections
Radiation source: fine-focus sealed tube	2534 reflections with *I* > 2.0σ(*I*)
graphite	*R*_int_ = 0.027
ω scans	θ_max_ = 28.8°, θ_min_ = 2.2°
Absorption correction: multi-scan (*SADABS*; Sheldrick, 2003)	*h* = −7→8
*T*_min_ = 0.603, *T*_max_ = 0.674	*k* = −21→21
16039 measured reflections	*l* = −17→17

### Refinement

**Table:**

Refinement on *F*^2^	Primary atom site location: structure-invariant direct methods
Least-squares matrix: full	Secondary atom site location: difference Fourier map
*R*[*F*^2^ > 2σ(*F*^2^)] = 0.030	Hydrogen site location: inferred from neighbouring sites
*wR*(*F*^2^) = 0.075	H-atom parameters constrained
*S* = 1.02	*w* = 1/[σ^2^(*F*_o_^2^) + (0.0302*P*)^2^ + 1.0145*P*] where *P* = (*F*_o_^2^ + 2*F*_c_^2^)/3
3081 reflections	(Δ/σ)_max_ = 0.001
147 parameters	Δρ_max_ = 0.51 e Å^−^^3^
0 restraints	Δρ_min_ = −0.54 e Å^−^^3^

### Special details

**Table:**

Geometry. All e.s.d.'s (except the e.s.d. in the dihedral angle between two l.s. planes) are estimated using the full covariance matrix. The cell e.s.d.'s are taken into account individually in the estimation of e.s.d.'s in distances, angles and torsion angles; correlations between e.s.d.'s in cell parameters are only used when they are defined by crystal symmetry. An approximate (isotropic) treatment of cell e.s.d.'s is used for estimating e.s.d.'s involving l.s. planes.
Refinement. Refinement of *F*^2^ against ALL reflections. The weighted *R*-factor *wR* and goodness of fit *S* are based on *F*^2^, conventional *R*-factors *R* are based on *F*, with *F* set to zero for negative *F*^2^. The threshold expression of *F*^2^ > σ(*F*^2^) is used only for calculating *R*-factors(gt) *etc*. and is not relevant to the choice of reflections for refinement. *R*-factors based on *F*^2^ are statistically about twice as large as those based on *F*, and *R*- factors based on ALL data will be even larger.

### Fractional atomic coordinates and isotropic or equivalent isotropic displacement parameters (Å^2^)

**Table:**

	*x*	*y*	*z*	*U*_iso_*/*U*_eq_	
Cu1	0.95537 (5)	0.835509 (17)	0.19694 (2)	0.03082 (9)	
Cl1	0.77693 (11)	0.94146 (4)	0.23143 (6)	0.04312 (15)	
Cl2	1.30421 (10)	0.81769 (4)	0.38962 (5)	0.04150 (15)	
O1	1.0897 (3)	0.90737 (10)	0.11255 (15)	0.0412 (4)	
N1	1.1191 (3)	0.77854 (12)	0.04581 (16)	0.0331 (4)	
H1	1.1581	0.7462	0.0018	0.040*	
N2	1.0103 (3)	0.74896 (11)	0.10466 (15)	0.0293 (4)	
N3	0.7804 (3)	0.73440 (12)	0.21178 (16)	0.0324 (4)	
C1	1.2447 (5)	0.90327 (18)	−0.0185 (2)	0.0453 (6)	
H1A	1.3934	0.9294	0.0333	0.068*	
H1B	1.1393	0.9470	−0.0699	0.068*	
H1C	1.2651	0.8590	−0.0656	0.068*	
C2	1.1484 (4)	0.86451 (15)	0.05182 (19)	0.0335 (5)	
C3	0.9627 (5)	0.60109 (15)	0.0319 (2)	0.0457 (6)	
H3A	1.0562	0.6197	−0.0033	0.069*	
H3B	0.8111	0.5834	−0.0295	0.069*	
H3C	1.0380	0.5529	0.0842	0.069*	
C4	0.9369 (4)	0.67248 (13)	0.09883 (18)	0.0304 (4)	
C5	0.8118 (4)	0.66154 (14)	0.16587 (19)	0.0309 (4)	
C6	0.7298 (4)	0.58379 (16)	0.1791 (2)	0.0424 (6)	
H6	0.7534	0.5334	0.1460	0.051*	
C7	0.6118 (5)	0.58128 (19)	0.2426 (3)	0.0532 (7)	
H7	0.5566	0.5286	0.2551	0.064*	
C8	0.5757 (5)	0.6552 (2)	0.2867 (3)	0.0529 (7)	
H8	0.4916	0.6546	0.3282	0.063*	
C9	0.6626 (4)	0.73087 (18)	0.2702 (2)	0.0425 (6)	
H9	0.6377	0.7820	0.3015	0.051*	

### Atomic displacement parameters (Å^2^)

**Table:**

	*U*^11^	*U*^22^	*U*^33^	*U*^12^	*U*^13^	*U*^23^
Cu1	0.03910 (16)	0.02660 (14)	0.03380 (15)	0.00029 (11)	0.02316 (12)	−0.00381 (11)
Cl1	0.0510 (4)	0.0394 (3)	0.0457 (3)	0.0123 (3)	0.0286 (3)	−0.0023 (2)
Cl2	0.0372 (3)	0.0551 (4)	0.0348 (3)	−0.0009 (3)	0.0194 (2)	0.0016 (3)
O1	0.0604 (11)	0.0295 (8)	0.0477 (10)	−0.0026 (7)	0.0372 (9)	−0.0024 (7)
N1	0.0447 (11)	0.0317 (9)	0.0341 (10)	0.0001 (8)	0.0280 (9)	−0.0009 (8)
N2	0.0362 (9)	0.0288 (9)	0.0295 (9)	0.0012 (7)	0.0211 (8)	−0.0011 (7)
N3	0.0328 (9)	0.0363 (10)	0.0328 (9)	−0.0008 (8)	0.0196 (8)	−0.0005 (8)
C1	0.0559 (16)	0.0463 (14)	0.0428 (13)	−0.0087 (12)	0.0310 (12)	0.0032 (11)
C2	0.0381 (12)	0.0335 (11)	0.0308 (11)	−0.0005 (9)	0.0180 (9)	0.0019 (9)
C3	0.0772 (19)	0.0281 (11)	0.0453 (14)	−0.0010 (12)	0.0402 (14)	−0.0053 (10)
C4	0.0382 (11)	0.0272 (10)	0.0275 (10)	0.0020 (9)	0.0173 (9)	0.0001 (8)
C5	0.0311 (10)	0.0319 (11)	0.0285 (10)	−0.0003 (9)	0.0134 (8)	0.0004 (9)
C6	0.0414 (13)	0.0364 (13)	0.0509 (14)	−0.0033 (10)	0.0234 (11)	0.0045 (11)
C7	0.0456 (15)	0.0498 (16)	0.0685 (19)	−0.0052 (12)	0.0309 (14)	0.0175 (14)
C8	0.0440 (15)	0.067 (2)	0.0615 (18)	0.0006 (13)	0.0366 (14)	0.0145 (15)
C9	0.0393 (13)	0.0541 (16)	0.0443 (14)	0.0026 (11)	0.0282 (11)	0.0022 (12)

### Geometric parameters (Å, °)

**Table:**

Cu1—N2	1.9653 (18)	C1—H1C	0.9800
Cu1—N3	2.0305 (19)	C3—C4	1.482 (3)
Cu1—O1	2.0592 (17)	C3—H3A	0.9800
Cu1—Cl1	2.2110 (7)	C3—H3B	0.9800
Cu1—Cl2	2.4892 (9)	C3—H3C	0.9800
O1—C2	1.240 (3)	C4—C5	1.482 (3)
N1—C2	1.359 (3)	C5—C6	1.380 (3)
N1—N2	1.367 (3)	C6—C7	1.392 (4)
N1—H1	0.8900	C6—H6	0.9500
N2—C4	1.283 (3)	C7—C8	1.367 (4)
N3—C9	1.334 (3)	C7—H7	0.9500
N3—C5	1.353 (3)	C8—C9	1.381 (4)
C1—C2	1.483 (3)	C8—H8	0.9500
C1—H1A	0.9800	C9—H9	0.9500
C1—H1B	0.9800		
			
N2—Cu1—N3	78.73 (8)	O1—C2—C1	122.7 (2)
N2—Cu1—O1	77.90 (7)	N1—C2—C1	117.6 (2)
N3—Cu1—O1	153.54 (7)	C4—C3—H3A	109.5
N2—Cu1—Cl1	157.16 (6)	C4—C3—H3B	109.5
N3—Cu1—Cl1	100.28 (6)	H3A—C3—H3B	109.5
O1—Cu1—Cl1	96.47 (5)	C4—C3—H3C	109.5
N2—Cu1—Cl2	100.84 (6)	H3A—C3—H3C	109.5
N3—Cu1—Cl2	96.46 (6)	H3B—C3—H3C	109.5
O1—Cu1—Cl2	99.95 (6)	N2—C4—C3	126.3 (2)
Cl1—Cu1—Cl2	101.94 (3)	N2—C4—C5	112.26 (19)
C2—O1—Cu1	113.58 (15)	C3—C4—C5	121.4 (2)
C2—N1—N2	113.82 (18)	N3—C5—C6	122.3 (2)
C2—N1—H1	121.4	N3—C5—C4	114.61 (19)
N2—N1—H1	124.6	C6—C5—C4	123.0 (2)
C4—N2—N1	124.94 (19)	C5—C6—C7	118.1 (3)
C4—N2—Cu1	120.27 (15)	C5—C6—H6	121.0
N1—N2—Cu1	114.72 (14)	C7—C6—H6	121.0
C9—N3—C5	118.6 (2)	C8—C7—C6	119.5 (3)
C9—N3—Cu1	127.53 (18)	C8—C7—H7	120.2
C5—N3—Cu1	113.48 (15)	C6—C7—H7	120.2
C2—C1—H1A	109.5	C7—C8—C9	119.4 (3)
C2—C1—H1B	109.5	C7—C8—H8	120.3
H1A—C1—H1B	109.5	C9—C8—H8	120.3
C2—C1—H1C	109.5	N3—C9—C8	122.0 (3)
H1A—C1—H1C	109.5	N3—C9—H9	119.0
H1B—C1—H1C	109.5	C8—C9—H9	119.0
O1—C2—N1	119.6 (2)		
			
N2—Cu1—O1—C2	−3.14 (17)	Cu1—O1—C2—C1	178.65 (18)
N3—Cu1—O1—C2	−31.6 (3)	N2—N1—C2—O1	4.0 (3)
Cl1—Cu1—O1—C2	−160.69 (16)	N2—N1—C2—C1	−174.3 (2)
Cl2—Cu1—O1—C2	95.94 (17)	N1—N2—C4—C3	2.5 (4)
C2—N1—N2—C4	170.3 (2)	Cu1—N2—C4—C3	179.18 (19)
C2—N1—N2—Cu1	−6.6 (2)	N1—N2—C4—C5	−175.88 (19)
N3—Cu1—N2—C4	−4.33 (17)	Cu1—N2—C4—C5	0.8 (3)
O1—Cu1—N2—C4	−171.84 (19)	C9—N3—C5—C6	−1.2 (3)
Cl1—Cu1—N2—C4	−94.1 (2)	Cu1—N3—C5—C6	172.25 (18)
Cl2—Cu1—N2—C4	90.18 (17)	C9—N3—C5—C4	177.8 (2)
N3—Cu1—N2—N1	172.68 (16)	Cu1—N3—C5—C4	−8.8 (2)
O1—Cu1—N2—N1	5.18 (14)	N2—C4—C5—N3	5.4 (3)
Cl1—Cu1—N2—N1	82.9 (2)	C3—C4—C5—N3	−173.0 (2)
Cl2—Cu1—N2—N1	−92.80 (14)	N2—C4—C5—C6	−175.6 (2)
N2—Cu1—N3—C9	179.8 (2)	C3—C4—C5—C6	5.9 (4)
O1—Cu1—N3—C9	−151.88 (19)	N3—C5—C6—C7	−0.2 (4)
Cl1—Cu1—N3—C9	−23.5 (2)	C4—C5—C6—C7	−179.1 (2)
Cl2—Cu1—N3—C9	80.0 (2)	C5—C6—C7—C8	1.7 (4)
N2—Cu1—N3—C5	7.08 (15)	C6—C7—C8—C9	−1.8 (4)
O1—Cu1—N3—C5	35.4 (3)	C5—N3—C9—C8	1.1 (4)
Cl1—Cu1—N3—C5	163.84 (14)	Cu1—N3—C9—C8	−171.3 (2)
Cl2—Cu1—N3—C5	−92.74 (15)	C7—C8—C9—N3	0.4 (4)
Cu1—O1—C2—N1	0.5 (3)		

### Hydrogen-bond geometry (Å, °)

**Table:**

*D*—H···*A*	*D*—H	H···*A*	*D*···*A*	*D*—H···*A*
N1—H1···Cl2^i^	0.89	2.34	3.226 (2)	170
C1—H1A···Cl1^ii^	0.98	2.63	3.529 (3)	153
C3—H3A···N1	0.98	2.56	2.945 (3)	104
C3—H3A···Cl2^i^	0.98	2.81	3.785 (3)	176
C3—H3C···Cl1^iii^	0.98	2.75	3.703 (3)	165
C7—H7···Cl1^iv^	0.95	2.68	3.529 (3)	149

Symmetry codes: (i) *x*, −*y*+3/2, *z*−1/2; (ii) *x*+1, *y*, *z*; (iii) −*x*+2, *y*−1/2, −*z*+1/2; (iv) −*x*+1, *y*−1/2, −*z*+1/2.
